# The premonitory phase of migraine is due to hypothalamic dysfunction: revisiting the evidence

**DOI:** 10.1186/s10194-022-01518-5

**Published:** 2022-12-13

**Authors:** Cedric Gollion, Roberto De Icco, David W. Dodick, Hakan Ashina

**Affiliations:** 1 Danish Headache Center, Department of Neurology, Rigshospitalet, Faculty of Health and Medical Sciences, University of Copenhagen, Copenhagen, Denmark; 2 Department of Neurology, University Hospital of Toulouse, Toulouse, France; 3 Department of Brain and Behavioral Science, University of Pavia, Pavia, Italy; 4 Headache Science & Neurorehabilitation Center, IRCCS Mondino Foundation, Pavia, Italy; 5 Department of Neurology, Mayo Clinic, Scottsdale, AZ USA; 6 Department of Clinical Medicine, Faculty of Health Sciences, University of Copenhagen, Copenhagen, Denmark; 7 Department of Neurorehabilitation / Traumatic Brain Injury, Rigshospitalet, Copenhagen, Denmark; 8 Department of Anesthesia, Critical Care and Pain Medicine, Beth Israel Deaconess Medical Center, Harvard Medical School, Boston, MA USA

**Keywords:** Prodromes, Pathophysiology, Headache disorders, Orexins, Neuropeptide Y, Dopamine

## Abstract

**Objective:**

To critically appraise the evidence for and against premonitory symptoms in migraine being due to hypothalamic dysfunction.

**Discussion:**

Some premonitory symptoms (e.g. fatigue, mood changes, yawning, and food craving) are associated with the physiologic effects of neurotransmitters such as orexins, neuropeptide Y, and dopamine; all of which are expressed in hypothalamic neurons. In rodents, electrophysiologic recordings have shown that these neurotransmitters modulate nociceptive transmission at the level of second-order neurons in the trigeminocervical complex (TCC). Additional insights have been gained from neuroimaging studies that report hypothalamic activation during the premonitory phase of migraine. However, the available evidence is limited by methodologic issues, inconsistent reporting, and a lack of adherence to ICHD definitions of premonitory symptoms (or prodromes) in human experimental studies.

**Conclusions:**

The current trend to accept that premonitory symptoms are due to hypothalamic dysfunction might be premature. More rigorously designed studies are needed to ascertain whether the neurobiologic basis of premonitory symptoms is due to hypothalamic dysfunction or rather reflects modulatory input to the trigeminovascular system from several cortical and subcortical areas. On a final note, the available epidemiologic data raises questions as to whether the existence of premonitory symptoms and even more so a distinct premonitory phase is a true migraine phenomenon.

**Graphical Abstract:**

Video recording of the debate held at the 1st International Conference on Advances in Migraine Sciences (ICAMS 2022, Copenhagen, Denmark) is available at: https://www.youtube.com/watch?v=d4Y2x0Hr4Q8. 
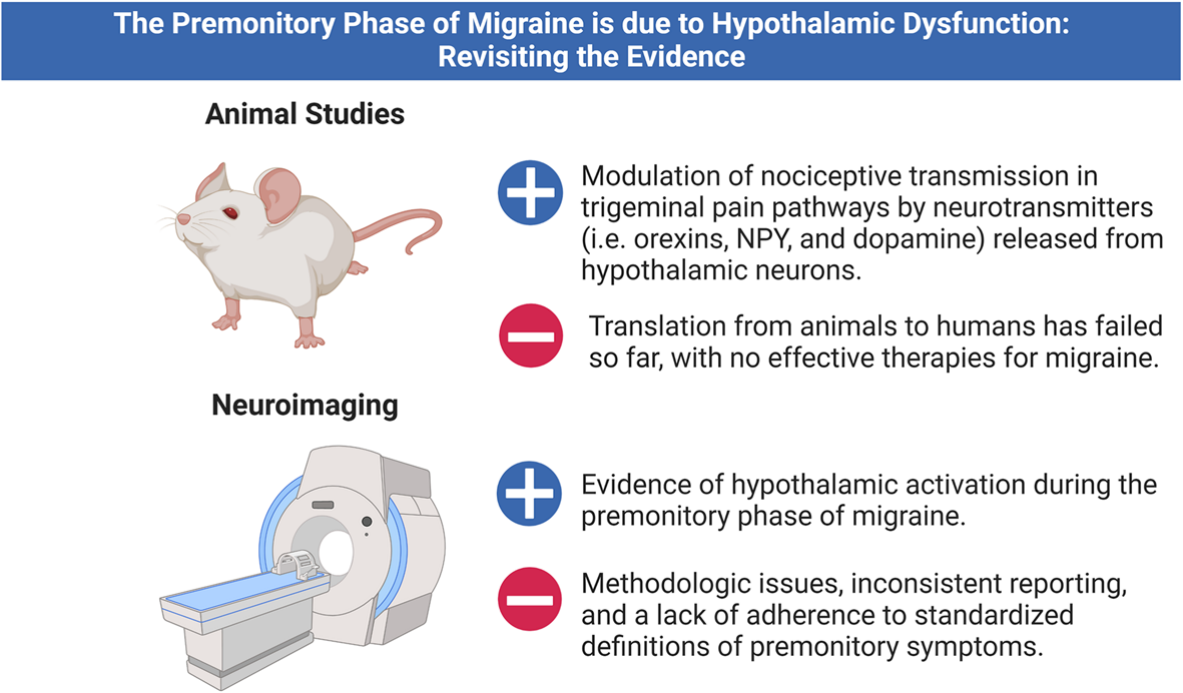

## Introduction

Migraine is a disabling neurologic disorder that afflicts more than one billion people worldwide [1]. The presenting feature of a migraine attack traditionally refers to aura or headache [2]. Some affected individuals do, however, experience premonitory symptoms that precede the onset of aura in migraine with aura or headache in migraine without aura [2]. These symptoms include fatigue, neck stiffness, and mood change while yawning and food craving is less common [3–20].

As migraine attacks are often appreciated through the description of distinct phases [21, 22] the term ‘premonitory phase’ is increasingly being used in academic literature [23]. It is nonetheless difficult to demark the exact beginning and end of the premonitory phase. Furthermore, the clinical assessment of premonitory symptoms in a patient is subject to recall bias and false attribution [24]. Critics therefore argue that further investigation is needed before the concept of a premonitory phase is widely adopted in clinical practice and research.

The neurobiologic basis of premonitory symptoms is of current interest, in part because of its possible relation to hypothalamic dysfunction [25]. A better understanding of the premonitory phase might, in turn, bring us closer to developing novel therapies that can prevent migraine attacks from being generated or terminate them before they evolve into subsequent phases. However, the current trend to accept the link between premonitory symptoms and hypothalamic dysfunction might be premature. A growing body of evidence from animal models, positron emission tomography (PET), and magnetic resonance imaging (MRI) have so far produced somewhat conflicting findings [26]. In this Review, we will therefore call attention to evidence for and against the premonitory phase of migraine being due to hypothalamic dysfunction.

## Terminology, definitions, and epidemiologic observations

The terms ‘premonitory symptoms’ and ‘prodromes’ are considered synonyms by most clinicians and researchers. The preferred term has changed over time, with the first three iterations of the International Classification of Headache Disorders (ICHD) endorsing use of the term premonitory symptoms [27–29], while the most recent iteration, ICHD-3, recommends the term prodromes [2]. The length of the premonitory phase is also debated, although most experts define it as a symptomatic phase that occurs up to 48 hours before the onset of aura or headache in a migraine attack [2]. In the following, we will use the term ‘premonitory symptoms’ for the purposes of consistency.

The presence of at least one premonitory symptom is generally considered sufficient to define a premonitory phase in an individual with migraine. However, there is no consensus on which specific symptoms should be classified as premonitory. This precludes accurate epidemiologic investigations and hinders comparative assessments. For example, four population-based studies have investigated the prevalence of premonitory symptoms in adults with migraine [3–6]. The reported prevalence ranged from 7.8% to 67.4% across the studies, and the corresponding figures are similarly discordant in clinical samples [7–18]. The incongruent epidemiologic data likely reflect differences in methodology and inconsistent definitions of premonitory symptoms [24]. It should also be noted that the presence of at least one premonitory symptom is sufficient to define a premonitory phase in most, if not all, observational studies [3–18]. With all these caveats, readers are encouraged to interpret the following evidence with caution.

In clinical samples [7–19], the most prevalent premonitory symptoms include fatigue, neck stiffness, mood change, and concentration difficulties. Less common are yawning, symptoms of depression, irritability, and food craving [7–19]. Some people also tend to report nausea, photo-, and phonophobia as premonitory symptoms [7–18]. The latter are, however, characteristic accompanying symptoms to the headache phase of a migraine attack [2], and it might be somewhat problematic to denote them as part of the premonitory phase if they occur within minutes before the onset of headache. However, the maintenance of symptoms through other phases of migraine does not negate their onset prior to the headache phase.

## Critical appraisal of neurotransmitter involvement

Although some premonitory symptoms are thought not to reflect hypothalamic dysfunction, others do provide a possible link in favor of this assertion. Indeed, fatigue, mood changes, yawning, and food craving have been associated with the physiologic effects of neurotransmitters such as orexins, neuropeptide Y (NPY), and dopamine; all of which are expressed in hypothalamic neurons [26, 30, 31]. Several animal experiments have supported the link between these neurotransmitters and the control of trigeminal pain [32–42]. However, the translation of these findings to humans has not led to a definitive conclusion. In this section, we discuss the evidence linking hypothalamic neurotransmitter to cephalic pain, provided by animal and human studies.

### Orexins

Orexin is a neuropeptide that, upon release from hypothalamic neurons, regulates wakefulness and appetite. It is posited to promote premonitory symptoms such as fatigue and food craving [30], and it exists in two isoforms, orexin A and B; both of which mediate their effects via binding to G protein-coupled receptors, i.e. the orexin 1 receptor (OX1R) and orexin 2 receptor (OX2R), [30]. Orexin A binds to OX1R and OX2R with similar affinity, whereas orexin B has a 10-fold greater affinity for OX2R, compared with OX1R [30].

In relation to cephalic pain, orexinergic involvement has primarily been investigated in rodents [32–35]. A series of in vivo studies have applied electric stimulation to the dura mater and middle meningeal artery (MMA) to activate sensory afferents of the trigeminal ganglion [32–35]. A recording electrode has then been used to measure nociceptive responses of second-order neurons in the trigeminal nucleus caudalis (TNC). Using this approach, experimental data have found that orexin A inhibits pro-nociceptive responses of TNC neurons when administered by microinjection into the posterior hypothalamus as well as by intravenous infusion into the femoral vein [32, 33]. In contrast, microinjection of orexin B into the posterior hypothalamus enhances pro-nociceptive responses of TNC neurons [32], whilst intravenous infusion of orexin B has no effect [33]. Another important observation from these experiments was that pre-treatment with a selective OX1R antagonist blocks the inhibitory effects of intravenously administered orexin A on TNC neurons [33].

Another important line of research on orexinergic involvement in mechanisms underlying cephalic pain has been to apply electric stimulation to dilate the MMA of rodents in vivo and examine the vascular effects of orexin A and B [34]. These experiments have revealed that intravenous infusion of orexin A, and not orexin B, attenuated MMA dilation, and this effect can be blocked using a selective OX1R antagonist [34]. Of note, intravenous infusion of CGRP was shown to induce MMA dilation that remained unaffected by administration of orexin A [34]. The latter observation led the authors to suggest that orexin A inhibits the endogenous release of CGRP from sensory afferents and thus has no effect on CGRP from an exogeneous source, i.e. intravenous infusion [34].

Based on the above, increased attention has been paid to the therapeutic promise of orexin receptor antagonism for the treatment of migraine [35, 43]. Preclinical data has even shown that a dual antagonist of OX1R and OX2R attenuates MMA dilation and pro-nociceptive responses of TNC neurons following electric stimulation to the dura mater and MMA [35]. However, a randomized, double-blind, placebo-controlled trial has found that filorexant – a dual orexin receptor antagonist – was ineffective for prevention of episodic migraine [43]. The reduction in mean monthly migraine days from baseline to months 1 through 3 was 1.7 days with filorexant and 1.3 days with placebo. Thus, it seems evident that therapeutic benefits are unlikely to be achieved with dual orexinergic receptor antagonism. However, preclinical data suggest that selective OX1R antagonism might hold therapeutic promise and thus underscore the need for clinical testing in people with migraine [32].

### Neuropeptide Y

Neuropeptide Y (NPY) is expressed in hypothalamic neurons and thought to promote the premonitory symptoms of mood changes and food craving [31]. Its G protein-coupled receptors are present at multiple levels of the trigeminovascular system, including on the vascular smooth muscle cells of intracranial arteries as well as first- and second-order trigeminovascular neurons [36, 44–46]. Early evidence suggestive of NPY involvement in cephalic pain mechanisms came from an in vitro study [36], in which NPY was shown to constrict intracranial arteries from human samples. A subsequent in vivo study then showed that pre-treatment with intravenously administered NPY attenuated plasma protein extravasation of meningeal vessels induced by electric stimulation of the trigeminal ganglion in rodents [37]. The same study also found that pre-treatment with NPY could inhibit plasma protein extravasation of meningeal vessels that had been induced by intravenously administered capsaicin [37]. More recent in vivo experimental data suggest that NPY can inhibit pro-nociceptive responses of second-order neurons in the trigeminal cervical complex of rodents following electric stimulation of the dura mater adjacent to the MMA [38]. Altogether, these findings support the assertion that NPY might inhibit nociceptive transmission in trigeminal pain pathways. However, clinical trials are warranted to ascertain the therapeutic promise of targeting NPY signaling in migraine.

### Dopamine

Dopamine is a neurotransmitter that is expressed in hypothalamic neurons [26, 47], and it has been posited to promote premonitory symptoms such as fatigue, nausea, and yawning [26]. The physiologic effects of dopamine are mediated through binding to its G protein-coupled receptors, of which there are five subtypes, labeled from D1 to D5 [39]. In rodents, the expression of D1, D2, D4, and D5 receptors have been shown in second-order neurons of the trigeminal nucleus caudalis and upper cervical spinal cord, i.e. trigeminocervical complex (TCC), [39].

In regard to cephalic pain, dopaminergic involvement has been studied mainly in rodents [39–42]. An important line of in vivo experiments provided early evidence that indicated a possible role of dopaminergic signaling in inhibition of pro-nociceptive responses at the level of TCC [39–41]. The authors first identified the presence of D1 and D2 receptors in the TCC of rodents using immunohistochemistry. The density of D2 receptors was found to be much higher than D1 receptors [41]. Following this, electric stimulation was applied to dilate the MMA which, in turn, increases the firing rate of TCC neurons. Dopamine was then administered by intravenous infusion into the femoral vein and microiontophoretic injection onto the TCC in separate experiments. The authors found that intravenous infusion of dopamine did not affect the firing rate of TCC neurons, whereas microiontophoretic injection of dopamine inhibited the firing rate [41]. Based on these observations, the same lab proceeded to examine the in vivo effects of various dopamine receptors agonists and antagonists on the baseline firing rate of TCC neurons in rodents after electric stimulation of the MMA [40]. The firing rate was found to be inhibited by intravenous infusion of quinpirole hydrochloride which is a selective D2 receptor agonist that can cross the blood-brain barrier (BBB) and act on receptors within the central nervous system [40]. Conversely, intravenous infusion of remoxipride hydrochloride (a selective D2-like receptor antagonist) and eticlopride hydrochloride (a selective D2/D3 receptor antagonist) that both can cross the BBB facilitated the firing rate of TCC neurons [40]. An additional observation was that the firing rate remained unaffected by intravenous infusion of domperidone (a D2-like receptor antagonist) alone which does not readily cross the BBB [40]. Moreover, it was reported that intravenous infusion of selective D1-like agonists and antagonists did not affect the firing rate of TCC neurons following electric stimulation of the MMA. Taken together, the authors concluded that dopamine is likely to exert its anti-nociceptive effects via binding to its D2 receptors in TCC neurons.

Another important line of research has focused on the physiologic effects of projections from dopaminergic A11 neurons in the posterior hypothalamus to neurons in the TCC of rodents [39, 42]. In one in vivo animal study [39], electric stimulation of A11 neurons was shown to attenuate neuronal firing in the TCC evoked by electric stimulation of the dura mater. This inhibitory effect was interestingly abolished following intravenous infusion of a selective D2/D3 receptor antagonist. Conversely, electric lesioning of A11 neurons resulted in facilitation of evoked neuronal firing in the TCC. The latter observation has since been challenged by the results of another in vivo animal study [42], in which partial chemical lesioning of A11 neurons inhibited pro-nociceptive responses at the level of second-order trigeminal neurons.

The therapeutic promise of targeting dopaminergic signaling in migraine has been examined in several clinical trials, but the results have been somewhat disappointing. In one randomized, double-blind, placebo-controlled, dose-ranging, multicenter trial, 305 adult participants with migraine were randomly allocated to receive intramuscular injection of droperidol (a D2 receptor antagonist) or placebo [48]. The authors found that 2.75 mg, 5.5 mg, and 8.75 mg of droperidol were superior to placebo in terms of pain freedom by 2 hours after drug administration. However, these doses of droperidol were poorly tolerated, with very common adverse events including akathisia, anxiety, asthenia, and somnolence. Droperidol is therefore not considered a viable therapeutic option for the treatment of acute migraine attacks.

Metoclopramide is a prokinetic antiemetic that exerts antagonistic effects on the D2 receptor and has been evaluated for the acute treatment of migraine [49, 50]. A 2004 meta-analysis concluded that parenteral administration of metoclopramide is seemingly effective for the acute treatment of migraine, albeit the included studies were limited by small samples and thus underpowered [49]. Also, addition of oral metoclopramide to acetylsalicylic acid has minimal effects on pain relief but provides substantial benefits in terms of relief of nausea and vomiting [51]. The present consensus appears to be that oral metoclopramide as well as oral domperidone – another antiemetic and D2 receptor antagonist – can be used as adjuncts to non-steroidal anti-inflammatory drugs and triptans in patients for whom migraine attacks are accompanied by nausea and/or vomiting [51].

## Critical appraisal of the imaging evidence

Neuroimaging is increasingly being used as a tool for investigating the origins of the premonitory phase of migraine [26]. The first piece of evidence of hypothalamic involvement in the premonitory phase came from a H_2_^15^O PET study, in which changes in cerebral blood flow – a surrogate marker of neuronal activation – were examined during spontaneous attacks in seven patients with episodic migraine without aura [52]. Additional scans were also performed after headache relief by sumatriptan injection and again during the interictal phase of migraine. The authors found increased regional blood flow in the hypothalamus and certain areas of the brain stem during spontaneous migraine attacks, compared with the interictal phase. It merits emphasis that these changes in regional blood flow persisted following headache relief by sumatriptan injection. Based on these results, the authors suggested that hypothalamic activation initiates the premonitory phase of migraine and modulates nociceptive transmission within the trigeminovascular system. However, it should be noted that the authors did not report whether any of the patients experienced premonitory symptoms, and no scans were performed during the premonitory phase of migraine, as patients were required to be at least 48 hours free of headache before and after the interictal scan session.

More recent evidence of hypothalamic involvement in the premonitory phase of migraine comes from a functional MRI study, in which one patient with episodic migraine without aura was scanned daily in the morning for a 30-day period [53]. The authors established their own case definitions for each phase of the ‘migraine cycle’. The pre-ictal phase was defined by onset of headache within the next 24 hours, while all days with headache were classified as the ictal phase. In addition, the interictal phase was defined as any time period occurring at least 60 hours before or after the ictal phase. During the 30-day scan period, the patient experienced three untreated migraine attacks, each of which were unilateral, lasted 1–2 days, and had peak pain headache of 5–7 on the visual analog scale. The experimental paradigm included visual stimuli using a rotating checkerboard and gaseous stimuli using ammonia (trigeminal nerve stimulation), rose odor (olfactory nerve stimulation), and air (control stimulation). The main study findings were increased activation within the hypothalamus and enhanced functional coupling between the hypothalamus and spinal trigeminal nuclei during the pre-ictal phase, as compared with the interictal phase. The authors also found enhanced functional coupling between the hypothalamus and dorsal rostral pons during the ictal phase when compared with the interictal phase. Taken together, the authors concluded that the hypothalamus is the “primary generator of migraine attacks”. In regard to the premonitory phase of migraine, it does merit emphasis that the authors did not report whether the patient experienced any premonitory symptoms. Further complicating the issue is the authors’ definition of the ictal phase as any day with headache, which does not meet the ICHD-3 criteria for a migraine attack. Careful considerations must therefore be made when interpreting these findings as evidence suggestive of hypothalamic involvement in the premonitory of migraine. It should, nonetheless, be noted that the same lab has since published another functional MRI study [54], in which seven patients with episodic migraine with or without aura were scanned daily for at least a 30-day period and underwent the same experimental paradigm as described above. The authors found hypothalamic activation to be present exclusively in the 48 hours preceding the onset of headache, i.e. the pre-ictal phase. No hypothalamic activation was thus observed during the headache phase of migraine or the post-ictal phase. Again, the authors did not provide any information about the presence or absence of premonitory symptoms in any of the scanned patients. This issue seems to be a consistent problem across neuroimaging studies in migraine [53–56], and a commitment to standardized data reporting and adherence to ICHD definitions should be a minimum requirement for future studies.

Another approach to investigate the association of hypothalamic dysfunction with premonitory symptoms is the combination of neuroimaging with experimental provocation of migraine attacks using the nitric oxide donor glyceryl trinitrate (GTN), [57–60]. This was first done in a H_2_^15^O PET study [57], in which eight patients with episodic migraine without aura and self-reported premonitory symptoms were included. All patients were required to be free of headache for at least 72 hours before the scans, and each of them were scanned at baseline, during the premonitory phase, and again during the headache phase of a GTN-induced migraine attack. Baseline scans were performed before the start of intravenous infusion of GTN. It should also be noted that premonitory scans were performed when the GTN-induced immediate headache had completely subsided, premonitory symptoms were present, and the onset of GTN-induced migraine had not occurred yet. Tiredness was the most frequent GTN-induced premonitory symptom (*n* = 5) followed by thirst (*n* = 4) and neck stiffness (*n* = 3). Compared with baseline, increased activation of the posterior hypothalamus was shown during the early premonitory phase (at the time of the first occurring premonitory symptom) but not in the late premonitory phase or during GTN-induced migraine. Of note, it should be mentioned that several cortical and subcortical structures other than the posterior hypothalamus were also shown to be activated during the early premonitory phase. Moreover, these findings should be interpreted with caution due to methodologic issues. First, stratification of premonitory symptoms into an early and late phase has, to our knowledge, not been reported previously. Second, the sample size was small, and two patients only had one premonitory scan because there was less than 15 minutes between the onset of the premonitory phase and the onset of GTN-induced migraine. Third, the authors did not include an active control group of patients with migraine and no premonitory symptoms or a control group of healthy volunteers free of headache. Lastly, it would have been useful to include a placebo comparator since the onset of premonitory symptoms might, in part, be attributed to nocebo effects.

Conflicting results have recently been published on hypothalamic involvement during the premonitory phase of GTN-induced migraine [58, 59]. In one randomized, double-blind, placebo-controlled, 2-way crossover, resting-state functional MRI study [58], the authors included 25 patients with migraine who had developed GTN-induced migraine preceded by at least 3 premonitory symptoms. These patients were then randomized to intravenous infusion of GTN or placebo on two separate experimental days. Scans were performed at baseline and at fixed time points corresponding to the time of onset of the premonitory and attack phase following the initial GTN infusion at screening. Compared with baseline, alterations in functional connectivity were reported in several areas (incl. The pons and thalamus) as well as in the thalamo-cortical network, albeit no changes were found in relation to the hypothalamus. These findings are nonetheless challenging to interpret since there were differences in functional connectivity at baseline between the two experimental days. Patients had also been received intravenous infusion with GTN during screening and were thus not naïve to GTN administration at the time of the subsequent 2-way crossover experiment, which raises the issue of spontaneous unblinding. Noteworthy, a thalamo-cortical dysfunction in the premonitory phase has been described by another group, but the involved cortical regions were different, and the observed findings are not comparable [61]. In another functional MRI study [59], the authors included 15 women with migraine without aura and 10 healthy women free of headache to evaluate the hypothalamic blood oxygen level–dependent (BOLD) response – a surrogate marker of neuronal activation – to oral ingestion of glucose on two separate days after overnight fasting. Patients with migraine had to be free of attacks for at least 3 days prior and 2 days after both experimental days. For both groups, the first day included scans at baseline and again after glucose ingestion, whereas all participants received intravenous infusion of GTN, orally ingested glucose, and were then scanned at 90 min after the start of infusion, i.e. premonitory scan. Twelve patients developed GTN-induced migraine, and these provoked attacks were all preceded by at least one premonitory symptom. In regard to the hypothalamic BOLD response, no differences were observed between patients with migraine and the control population after glucose ingestion on the first experimental day. The BOLD response to glucose ingestion was, however, significantly different between the second and first experimental day (GTN day versus non-GTN day) in patients with migraine but not in the control population. Furthermore, no differences were found in hypothalamic BOLD response to glucose ingestion after GTN infusion when comparing patients with migraine and healthy controls. On another note, the authors also scanned five patients with migraine during the premonitory phase of spontaneous attacks but found no differences in BOLD response to glucose ingestion between spontaneous and provoked attacks at the intra-individual level. The interpretive challenge here is the inconclusive findings, and the need for additional neuroimaging research is clear (Table 1).

**Table 1:** Imaging appraisal of hypothalamus dysfunction in the premonitory phase of migraine

First Author, Publication Year	Imaging Method	Study Population(s), Sample Size (n)	Definition of the Premonitory Phase	Main Findings	Limitations
Denuelle et al., 2007 [52]	H_2_^15^O PET	MO, *n* = 7	No case definition was provided, and the authors did not report whether the participants reported any premonitory symptoms.	Hypothalamic and brainstem activation within the first four hours of spontaneous migraine attacks, persisting after pain relief. The authors suggested hypothalamic involvement in non-painful symptoms.	Patients were not scanned during the premonitory phase. No control group.
Maniyar et al., 2014 [57]	H_2_^15^O PET	MO, *n* = 8	Period after cessation of unspecific NTG-induced headache and before migraine headache, when patients started to experience symptoms warning them of an impending headache. These symptoms had to be present on at least two enquiries (initially every 5 min and then every 10–15 minutes).	Hypothalamic activation during the early premonitory phase, but not in the late premonitory phase nor in the headache phase.	Hypothalamic activation was not observed when early and late premonitory phase were pooled together. Lack of definition of the early and late premonitory phase. No placebo control. No control group.
Maniyar et al., 2014 [60]	H_2_^15^O PET	MO, *n* = 10		Activation in rostral dorsal medulla and periaqueductal grey matter, but not in hypothalamus, in patients experiencing nausea.	Small sample size of patients with nausea (*n* = 3). No placebo control. No control group.
Maniyar et al., 2014 [73]	H_2_^15^O PET	MO, *n* = 10		Higher activation of extrastriate cortex in patients with photophobia during the premonitory phase of NTG-triggered attacks. No hypothalamic activation.	Small sample size of patients with photophobia (*n* = 5) No placebo control. No control group.
Schulte et al., 2016 [53]	BOLD fMRI	MO, *n* = 1	No case definition was provided, and the authors did not report whether the participants reported any premonitory symptoms.	Activation of hypothalamus in the pre-ictal phase. Functional coupling of hypothalamus with spinal trigeminal nucleus, which switch to dorsal pons in the headache phase.	Premonitory symptoms were not reported. Results corresponded to the preictal phase, not the premonitory phase, per se. No control group.
Schulte et al., 2020 [54]	BOLD fMRI	MO, *n* = 6 MA, *n* = 1	No case definition was provided, and the authors did not report whether the participants reported any premonitory symptoms.	Hypothalamus activation started 48 hours prior to the headache phase.	
Meylakh et al., 2018 [56]	BOLD fMRI	MO, *n* = 21 MA, *n* = 5	No case definition was provided. The authors reported that the patients had no predicting factor that they were 24 hours prior to a migraine attack. No definition of the predicting factors.	Increased infra-slow oscillatory power only prior to a migraine attack in the brainstem, thalamus and hypothalamus. Increase functional connectivity between PAG and hypothalamus prior to the migraine attack.	Migraine phases were retrospectively defined and no predictive factors to be in the preictal phase, including premonitory symptoms, were reported. Overall, increased and decreased functional connectivity between hypothalamus and PAG.
Meylakh et al., 2020 [74]	ASL and BOLD fMRI	MO, *n* = 25 MA, *n* = 9	No case definition was provided. The authors reported that the patients had no predicting factor that they were 24 hours prior to a migraine attack. No definition of the predicting factors.	Decrease regional cerebral blood flow in the lateral hypothalamus and decrease functional connectivity of lateral hypothalamus with pain processing pathway, including PAG.	
Karsan et al., 2020 [58]	BOLD fMRI	MO, *n* = 10 MA, *n* = 15	Period with at least 3 symptoms typical for the subject, assessed by questionnaire, after NTG-infusion in the absence of any migraine headache, consistently reported on 2 separate episodes of enquiry.	Several changes in connectivity of thalamus with precuneus and cuneus, pons with limbic lobe, cingulate and frontal cortices in the premonitory phase.	Connectivity of hypothalamus was assessed but not its activity/response to external stimuli. Baseline hypothalamic connectivity was not comparable between active and placebo groups.
Van Oosterhout et al., 2021 [59]	BOLD fMRI	MO, *n* = 15	Period with at least 1 symptom assessed by questionnaire after NTG-infusion before the onset of a provoked migrainous headache.	Hypothalamic signal recovery phase was abnormally faster and steeper in the premonitory phase of a NTG-triggered and spontaneous attacks, suggesting hypothalamic dysregulation.	Dysregulation was observed in response to a specific hypothalamic activation, not during its spontaneous activity. No placebo control.

*ASL* Arterial Spin Labelling. *BOLD* Blood Oxygen Level Dependent. *CBF* Cerebral Blood Flow. *fMRI* functional Magnetic Resonance Imaging. *HC* Healthy Controls. *MA* Migraine with Aura. *MO* Migraine without Aura. *NTG* Nitroglycerin. *PAG* Periaqueductal Grey Matter. *PET* Positron Emission Tomography

## Lessons learned and future directions

As we collectively work toward a better understanding of hypothalamic involvement during the premonitory phase of migraine, it becomes important to summarize what lessons can be learned and what steps must be taken in the future.

The available evidence from animal studies suggests that specific signaling molecules (i.e. orexins, NPY, and dopamine) released by hypothalamic neurons modulate nociceptive transmission at the level of second-order trigeminal neurons [32–42, 47]. An important next step will be to record the effect of these signaling molecules on the firing rate of first-order neurons in the trigeminal ganglion and third-order neurons in the thalamus. In addition, prospective sampling of blood and cerebrospinal fluid can be used to examine whether concentrations of orexins, NPY, and dopamine differ between the premonitory, ictal, postdromal, and interictal phase of migraine.

Insights from neuroimaging studies are currently limited by the lack of adherence to ICHD definitions of premonitory symptoms (or prodromes), [53–57, 62]. Further complicating the matter is the inconsistent reporting of whether study participants did experience any premonitory symptoms at the time of the scan [53–56, 62]. It must be stressed that the premonitory phase is defined as a symptomatic phase, and it is incorrect to use this term if study participants are asymptomatic. Instead, some have used the term pre-ictal phase to describe a symptomatic or asymptomatic phase preceding the onset of a migraine attack (with or without aura) by up to 72 hours. However, the ICHD does not provide recognize the pre-ictal phase as a distinct entity, and an expert consensus on this matter seems warranted. On another note, available neuroimaging studies have largely focused on assessing the premonitory phase in relation to the hypothalamus. This approach might be too simplistic, as some premonitory symptoms are thought not to reflect hypothalamic dysfunction, and other cortical and subcortical structures have been implicated in migraine pathogenesis [53–58, 60, 62, 63]. Thus, it seems reasonable to suggest that several areas of the brain might be involved in modulation of nociceptive transmission within the trigeminovascular system. On a final note, neuroimaging studies might benefit from instructing participants to record prospectively the occurrence of premonitory symptoms ahead of the scan(s).

A growing number of studies are using the human provocation model to induce premonitory symptoms and migraine attacks using a pharmacologic agent, mainly GTN [57–60, 64–66]. This approach is limited by several methodologic issues that might be difficult to fully address even when the study is rigorously designed. For example, intravenous infusion of GTN induces most often a biphasic headache response in people with migraine [67]. The initial mild headache occurs almost immediately and tends to attenuate in some or resolve completely in others before the onset of the delayed headache fulfilling the criteria for a provoked migraine attack [67]. The premonitory phase is then usually defined as the symptomatic phase after the immediate headache has resolved completely and before the onset of the delayed migraine attack. This time period can be less than 15 minutes in some people with migraine [57], which, in turn, makes it challenging to investigate a distinct GTN-induced premonitory phase. Also, findings on neuroimaging might simply reflect changes related to the initial mild headache induced by GTN. Future studies are also encouraged to use control groups such as people with migraine who do not experience GTN-induced premonitory symptoms, people with tension-type headache, and healthy volunteers free of headache. This approach can facilitate ascertainment of whether observed changes in the hypothalamus are indeed specific to people with migraine who experience premonitory symptoms.

A final point of emphasis is the methodologic shortcomings that are evident in the epidemiologic literature [24]. Most observational studies have not adhered to ICHD definitions of premonitory symptoms (or prodromes), and about 100 premonitory symptoms have been described so far [3–18]. There is also epidemiologic data to suggest that symptoms labelled as premonitory are equally common in the headacheand postdromal phase of migraine. In people with a high frequency of migraine attacks, it might be difficult to determine whether a specific symptom should be labelled as premonitory or postdromal if the between-attack interval is not sufficient and since some of the symptoms that occur during the premonitory phase may also be seen in the interictal phase [10, 15, 68–71].. In addition, one population-based study reported that premonitory symptoms were no more frequent in migraine, compared with tension-type headache [72]. Collectively, questions can be raised as to whether the existence of premonitory symptoms and even more so a distinct premonitory phase is a true migraine phenomenon.

## Conclusions

Experimental studies have brought important information on basic processes underlying the premonitory phase of migraine. The available evidence seems to suggest that hypothalamic activation is the principal pathogenic driver of premonitory symptoms in migraine and can modulate nociceptive transmission within the trigeminovascular system. This notion might however be premature, as the available evidence is limited by methodologic issues and require replication in more rigorously designed studies. Further research is needed to first understand the epidemiologic patterns of premonitory symptoms in migraine and then to ascertain whether they truly reflect hypothalamic dysfunction.

## Data Availability

Not applicable.
